# A business process clustering algorithm using incremental covering arrays to explore search space and balanced Bayesian information criterion to evaluate quality of solutions

**DOI:** 10.1371/journal.pone.0217686

**Published:** 2019-06-13

**Authors:** Hugo Ordoñez, Jose Torres-Jimenez, Carlos Cobos, Armando Ordoñez, Enrique Herrera-Viedma, Gildardo Maldonado-Martinez

**Affiliations:** 1 Research Laboratory in Development of Software Engineering, Universidad de San Buenaventura, Cali, Valle del Cauca, Colombia; 2 Information Technology Laboratory, CINVESTAV-Tamaulipas. Cd. Victoria, Tamaulipas, México; 3 Information Technology Research Group (GTI), Universidad del Cauca, Popayán, Cauca, Colombia; 4 Intelligent Management Systems, University Foundation of Popayán, Popayán, Cauca, Colombia; 5 Andalusian Research Institute in Data Science and Computational Intelligence, Universidad de Granada, Granada, Spain; 6 Department of Electrical and Computer Engineering, Faculty of Engineering, King Abdulaziz University, Jeddah, Saudi Arabia; Bangladesh University of Engineering and Technology, BANGLADESH

## Abstract

The reuse of business processes (BPs) requires similarities between them to be suitably identified. Various approaches have been introduced to address this problem, but many of them feature a high computational cost and a low level of automation. This paper presents a clustering algorithm that groups business processes retrieved from a multimodal search system (based on textual and structural information). The algorithm is based on Incremental Covering Arrays (ICAs) with different alphabets to determine the possible number of groups to be created for each row of the ICA. The proposed algorithm also incorporates Balanced Bayesian Information Criterion to determine the optimal number of groups and the best solution for each query. Experimental evaluation shows that the use of ICAs with strength four (4) and different alphabets reduces the number of solutions needed to be evaluated and optimizes the number of clusters. The proposed algorithm outperforms other algorithms in various measures (precision, recall, and F-measure) by between 12% and 88%. Friedman and Wilcoxon non-parametric tests gave a 90–95% significance level to the obtained results. Better options of repository search for BPs help companies to reuse them. By thus reusing BPs, managers and analysts can more easily get to know the evolution and trajectory of the company processes, a situation that could be expected to lead to improved managerial and commercial decision making.

## Introduction

The daily activities and experiences of organizations are represented in Business Processes (BPs) comprising information about the interaction between systems and partners [1]. BPs are employed today in diverse fields including product manufacturing, service delivery, and inventory management [2]. BPs improve the resource management of an organization by encouraging reuse of existing processes [3]. Reuse of BPs thus offers a wealth of advantages, such as reductions in the time to market and the maintenance of existing processes. This reuse requires the identification of similarities between BPs and families of BPs, which in turn need to identify common tasks between these BPs [4]. Identifying similarities between BPs may become a cumbersome task as it involves the analysis of large volumes of data [5][6].

BPs are generally not static elements that belong to one single area or division within organizations. Rather, they are dynamic elements that may change over time, integrating various departments and/or employees. Modified BPs leads to the appearance of families of processes composed of similar BPs. The possibility of identifying these families enables analysts to make a systematic analysis of the BPs of an organization, thereby promoting reuse.

In this context, several research approaches have been presented for grouping BPs together according to their similarities. Most are based on measures such as textual information, structure, and behavior. Other information retrieval (IR) techniques have similarly been applied to improve the results of such research [7], including the multimodal approach [8].

For their part, clustering techniques aim at forming groups of BPs in accordance with common features such as structure, control flow, and tasks [9]. This grouping enables engineers and BPs experts to explore results in an organized way, making it easier to redesign and reuse BPs. In this vein, a clustering process is a crucial tool for improving the display and analysis of results [10,11].

Existing approaches organize the whole repository into groups or dendograms that simulate the organizational structure of the companies, meaning that users must search through these groups or organization charts. In contrast, the present approach provides the user with a set of BP clusters based on previously filtered results.

This paper presents ICAClusterBP, an algorithm for improving the visualization of results in a BP search system. This algorithm takes as input a set of BPs retrieved from a multimodal search component [6,12] and using Incremental Covering Arrays (ICAs) selects the best clustering solution [13]. Briefly, an ICA is a smart mechanism to sample complex multidimensional spaces (in this paper the ICA enables a smart sampling of the solution space to solve a BPs clustering problem). A list of ICAs with different alphabets is used to minimize the number of trials and increase the chance of finding better clusters in less time. Likewise, the algorithm allows selecting the best grouping according to Balanced Bayesian Information Criterion (BBIC) [14].

The present method comprises the activities:

- Determine the best distance measure within the grouping process. This measure makes it possible to know the closeness or similarity of the elements within each group, thus increasing the cohesion and compactness of the groups created.
- Calculate the lowest strength of ICA needed to obtain the desired performance, i.e., the size of the interaction between BPs in the clustering process.
- Identify the lowest alphabet of ICA required achieving the desired performance, i.e., determine the correct number of groups a solution must contain.

The present approach enables analysts to identify families or groups of BPs and analyze them to reuse these BPs in a new BP. Grouping similar BPs may also help analysts determine the most frequent changes in BPs. These changes may be due to process updates or other issues associated with market requirements.

Evaluation of the present approach was done using a BPs repository created collaboratively by experts [15]. It was further compared with the results of previous works [16] and other state-of-the-art algorithms. The proposed algorithm outperforms previous approaches in various measures (precision, recall, and F-measure) by between 12% and 88%. Friedman and Wilcoxon non-parametric tests gave a 90–95% significance level to the obtained results. The ICA-based process allows effective exploration of the entire universe of combinations for the possible groupings. Besides, by using BBIC it is possible to find a suitable number of groups and select the best solution evaluated.

The remainder of this paper is organized as follows: Section 2 presents related work. Section 3 provides a conceptual background on ICAs. Section 4 describes the proposed approach as well as an example of its use. Section 5 describes the process to determine the parameters of the algorithm. Evaluation of grouping quality is detailed in Section 6. Finally, Section 7 depicts conclusions and future work.

## Related work

Grouping of BPs first requires the selection of BPs that will make up the groups. Selection is based mainly on the similarity of BPs. These BPs are commonly represented as trees or graphs (with nodes and edges). The edit distance of the graph represents the set of operations needed to transform one BP into another. Based on this representation, Dijkman et al. [17] propose a method to measure the similarity of a query BP and a set of BPs in the repository, based on similarity of node labels. This method calculates the edit distance of the strings of the node labels of two BPs. The similarity between two graphs is defined as the sum of the label similarities. For their part, Malinova et al. [18] define BPs similarity as the ratio of the edit distance of the graph and the number of common elements between two BPs. The edit distance of the graph is defined as the number of operations (insert, delete nodes and edges) needed to transform one graph into another. For Aiolli et al. [19], similarity is based on node similarity and dependence similarity. Here, the nodes are modeled as vectors, and the cosine distance is used to calculate the similarity between these vectors.

Other approaches are based on linguistic, structural, and behavioral similarity of the BPs. Linguistic-based approaches analyze the name or the description of the activities, events, and logic gates. During the search process, some information retrieval techniques are used to create the ranking of relevant results. Those techniques include space-vector representation with a terms frequency (TF) and cosine distance value. In this group of techniques, inputs and/or outputs are matched based on linguistic or semantic information, leaving aside execution flow or behavior. Therefore, no account is taken of information regarding behavior patterns, activity type, and purpose of the task or activity [20,21].

Elsewhere, structure-based approaches analyze previous executions of BPs recorded in Log files. The search process detects phrases associated with BPs activities using domain ontologies. Activity patterns are similarly identified. The results list is created using a heuristic component that determines the frequency of the patterns detected. This group of techniques uses previous executions in log files and analyses nodes, connectors, and source code, among others. Among these techniques, data mining algorithms such as "Apriori" have been used to identify common elements between the BPs. It should be noted that these approaches depend on the execution time needed to perform the matching. In addition, various conditions must be fulfilled to obtain the association rules and guarantee the proper operation of this type of algorithms. These conditions include that the data in the logs must be well structured, and the most relevant information must be analyzed manually by experts (This implies that the user must have experience in BPs) [22,23].

Approaches based on similarity behavior meanwhile use genetic algorithms to transform BPs into a formal representation (e.g., graphs or state machines). Additional data can be included during the search process to provide higher precision, such as number of inputs and outputs per node, edge labels, node name or description. The execution time of these approaches is high because the algorithms must find a reference BP with the lowest value of the weighted distance to the BP in the repository. In these approaches, a fitness function is used to obtain an overall assessment that does not define how each part of the candidate model conforms to the reconstruction variants of the BP model to be discovered. These genetic algorithms start by reading the event logs, an activity which exponentially increases the order of the algorithm. The list of recovered BPs depends on the calculation of many variables since the dependence between activities must be analyzed. Meanwhile, the importance of each task is calculated considering only the number of times it appears in the log [24,25].

The grouping results of the above approaches can be improved by expanding the information features of the BP: activity description, task type, gate type, structure, and behavior, among others.

The reported works related to BPs clustering can be classified into four categories:

### Sequential clustering

Ferreira in [26] proposes a sequential grouping algorithm with the objective of organizing a series of objects in a set of groups, where each group contains objects that are similar for a type of measure. This measure depends on the type of objects or data present in the BP. Each group is associated with a probabilistic model, usually a Markov chain, similar to those presented by [27] and [28]. If the Markov chains are known for all the groups, then each input sequence is assigned to the group that can best produce the sequence. The algorithm carries out the following steps: 1) it initializes the models of each group (that is, the Markov chain for each group) at random; 2) it assigns each input sequence to the group that can produce the highest probability; 3) it estimates for each cluster model the series of sequences that belong to that group. Finally, steps 2 and 3 are repeated until the definitive cluster models are found.

Meanwhile, [29] and [21] propose a clustering approach that groups and identifies thematic topics present in the BP without the need to provide input information. Grouping was performed with the purpose of finding valuable information about the type of sequences that are running in BPs. The grouping procedure includes: an alpha algorithm, which is capable of recreating the BP of a Petri net according to the relationships found in the BP execution record; inference methods that consider the execution record as a simple sequence of symbols, inspired by the Markov model (identical to those presented by [30]) and that generate a graphical model that considers Markov chains of increasing order with non-cyclic directed graphs; a sequential clustering algorithm that takes into account a set of execution frameworks of the same process, which separates the traces into groups and finds the dependency diagram separately for each group; and a genetic algorithm that represents each solution using a causal matrix, that is, a map of the inputs and output dependencies for each activity.

These proposals present certain limitations, namely: they leave aside the flow of execution, they do not consider frequent pattern similarity, they use a random BP as a starting point to create groups, and finally, during the grouping process, task sequences that occur only once are eliminated.

### Hierarchical clustering

In [31] they present a BPs grouping scheme (as do [32] and [28]) for recovery of graphic schemes in similar groups of (sub) processes and their relationships. It starts with a macroprocess to finally get to the most specific activities, for which a set of directed graphs G_i_ = <N_i_, A_i_> is taken, where N_i_ is the set of nodes and A_i_ ⊆ N_i_ * N_i_ is the set of possibly labeled arcs, generating a grouping skeleton typical of substructures. The graphs are iteratively analyzed to discover in each step a group of isomorphic sub-structures. Clustering is used to compress the graphs, substituting each occurrence of the substructure with a node; this process is repeated until no further compression is possible.

Similarly, [33] present an alpha algorithm for clustering (as do [34]), which transforms BPs into two sets of relationships between their activities. The algorithm selects the perspectives that should be considered as relevant for the comparison. The aim is to convert a given BP into two sets: one set of relationships between the activities that must occur and another set of relationships that may or may not occur. The clustering algorithm links a similarity measure between two groups, which is defined as the similarity of all pairs of activities belonging to the two groups. The objective is to start with each of the elements of a single group and, in each iteration of the algorithm, two or more groups are merged as one. The algorithm is run until all the groups formed are merged into a single group that contains all elements with the greatest similarity. Once the final group is formed, it is plotted through a hierarchical structure in the form of a tree [35] called a dendrogram (commonly used in data mining).

In these methods, the search is based on such data as activity name, activity duration and number of errors, leaving out information on activity type and behavioral semantics. Neither are sequences (structural or behavioral information) considered in the course of the grouping process.

### Partitional clustering

Another technique used for grouping BPs models is *k-means*, which divides n observations into k groups represented by their centroids. It starts by defining a set of k random centroids and then iteratively assigns each observation to a cluster with the most similar centroid and after assigning all observations to their groups, updates the centroids that represent them. If a stop criterion is not met, the algorithm continues with the iterative loop. Otherwise, it returns the centroids of the clusters and the assignment of the observations to the groups. For a detailed description of the *k-means* algorithm see [36].

Qiao et al propose an approach to grouping and recovering business processes. The algorithm is based on the similarity of the flow of control represented in the structure and the description that represents the semantics present in the description of the BPs. To define the level of similarity, it uses a model called LDA-based retrieval [27]. Other works are related to the grouping of BPs from a repository of Log files with previous executions of BPs [37]. In these proposals, the algorithm groups BPs models with the same type of behavior based on the execution flow and the temporal dimension [38,39]. Some papers also relate the process of grouping with that of predicting the monitoring of the process, i.e. predicting the results of a case in run time (which may or may not be incomplete). To achieve this, the groups are formed based on the information on the trace registered in the Log files [40].

Elsewhere, Ordoñez et al. use *k-means* to group BPs retrieved from a repository through a multimodal search system that integrates textual and structural information [41]. Structural information is represented as codebooks in the form of text strings that simulate the formation of non-continuous and cumulative N-grams. Both the textual and structural information are stored in an array that simulates a vector space representation. *k-means* uses cosine similarity to calculate the similarity between the BPs that will be grouped. The elements are assigned to each group according to the degree of similarity of the textual and structural information. Melcher et al. also group BPs using the similarity of the behavior structure of BPs models. This structure is represented using vectors, and the algorithm uses Euclidean distance to determine the similarity of elements in each group [28].

A limitation of this type of method is that the number of groups must be established *a priori*. In addition, these algorithms have a high computational cost, as they are iteratively moving BPs between groups according to their similarity to the cluster centroid.

### Miscellaneous approaches for solving business process clustering

Ordoñez et al. presented an incremental algorithm that incorporates a multimodal search strategy to retrieve the BPs that will be grouped [8]. During the grouping, BPs are represented as vectors, and the similarity degree between vectors is calculated using a similarity function based on fuzzy logic. This similarity function determines the similarity degree between vectors based on the number of common elements between the BPs and the relevance of each term. In addition, Ordoñez, Corrales, and Cobos present a proposal that implements a multimodal search on a repository of BPs and the results are then grouped based on summaries (Fragments) with textual terms of the BPs retrieved in the query. The algorithm executes the following steps: identification of candidate phrases or terms with which to label the group, induction of group labels, identification of BPs that belong to each group, and final formation of the BPs groups [42].

Ordoñez, Torres et al propose a business process grouping scheme that, like the previous ones, implements a multimodal search strategy as the first stage for the recovery of the BPs that contains a degree of similarity with a query BP. This approach introduces improvements in the grouping phase by incorporating a Covering Array (to minimize the number of tests and maximize the possibility of finding good results) and an algorithm that determines the best grouping based on the lowest value of the sum of squares error (SSE) [16].

Against this background, it can be said that most of the existing search approaches only consider the similarity of labels and the position of the nodes. On the other hand, some approaches consider the semantic analysis of the node labels but depend on external elements such as Wordnet dictionary. The above approaches are limited to the matching of inputs and outputs using textual information of the BPs elements. Besides, these works leave aside execution flow, behavior, structure, activity type, gate type, and event type. Unlike previous works, multimodal approach [6,12] integrates textual information (such as task names and descriptions, events, and gates), and structural information. Structural information is defined by codebooks containing information of BPs behavior such as task-task, task-gate, task-event-gate. The multimodal approach offers a more extensive representation of the subject of study (BPs). Consequently, search options and relevance of search results are improved.

When only one element is considered—for example, the textual element—the grouping is carried out comparing the information of names and description of the BPs elements. Moreover, the created groups ignore BPs with similarities in structure, task types, and behavior.

On the other hand, in *k-means*-based approaches, the grouping has the disadvantage that a predefined number of groups is required. For its part, the grouping in the incremental algorithm starts with the assignation of the BPs to an initial group, and then the algorithm analyses the created groups and relocates the BPs to another group that crosses a pre-established similarity threshold. In this approach, BPs that fall short of the threshold are excluded from the grouping even though these are relevant to the interests of the user.

Unlike previous works, the present approach unifies the structural behavior units and textual features of BPs models in a single search space. With this representation, known as multimodal search representation, the search results are improved when the number of considered features is high (these features may be task description, task type, gate type, among others). Furthermore, in this approach, the BPs retrieved from a multimodal search system are grouped using a clustering algorithm. This clustering algorithm incorporates an ICA with different alphabets that allows determining the possible number of groups with each ICA. In turn, each ICA has a strength size t that determines the minimum interactions of BPs to achieve the best grouping. The proposed algorithm also incorporates BBIC [14,43] to determine the optimal number of groups for each query. The main difference with previous works lies in the fact that using ICAs with different alphabets reduces the number of evaluated solutions and improves the number of clusters created.

## Incremental covering arrays

The CA (*N; t*, *k*, *v*) is a mathematical object that has been successfully applied in diverse fields such as software and hardware testing [44]. The two most important features of a CA are Maximum Coverage and Minimal Cardinality. Maximum coverage means that in each submatrix of size *N* x *t* each of the *v*^*t*^ combinations appears at least once. Minimal cardinality indicates that the number of rows (tests) required that satisfy coverage is the minimum [45,46]. In a succinct way, a CA can be viewed as a smart sampling technique for multidimensional spaces. The following are the definitions considered for the incorporation of ICAs in the present work.

### Definition 1.1

A covering array (CA) is defined with the notation CA *(N; t*, *k*, *v)*. It is a matrix of size *N* by *k*, in which each element takes values from {*0*, *1*, *…*, *v-1*} and each *t* columns covers at least once the elements from *v*^*t*^. *N* indicates the number of rows (cardinality), *k* indicates the number of columns (degree), *v* is the alphabet (order), and *t* is the size of interaction (strength). A CA *(N; t*, *k*, *v)* can be used to sample the solutions of a clustering problem instance in the following way:

*N* is the number of possible clustering solutions that are sampled;
*k* is the number of elements to be grouped (each column represents an element to be grouped);
*v* is the maximum number of groups that will be formed;
*t* means that at least in one solution we sample each of the possible ways to group each *t* elements.

This way, if in the “i-th” row and “j-th” column the value “a” appears, this means that in the “*i*-th” solution sampled the “*j*-th” element belongs to the *a*-th group).

### Definition 1.2

An incremental covering array (ICA) [13,44] uses the notation ICA(*N*_*1*_, *N*_*2*_, *…*, *N*_*t*_*; t*, *k*, *v*) subject to *N*_*1*_*≤ N*_*2*_*≤…≤N*_*t*_ and satisfies CA(*N*_*1*_*; 1*, *k*, *v*),…, CA (*N*_*t*_*; t*, *k*, *v*) i.e. the first *N*_*i*_ rows form a covering array of strength *i*. The *t* CAs derived from an ICA (*N*_*1*_, *N*_*2*_, *…*, *N*_*t*_*; t*, *k*, *v*) satisfies CA (*N*_*1*_*; 1*, *k*, *v*) ⊆ CA (*N*_*2*_*; 2*, *k*, *v*) ⊆… ⊆ CA (*N*_*t*_*; t*, *k*, *v*) i.e., the *i*-th CA in the chain contains all the rows of the CAs with strength less than *i*.

**Fig 1:**
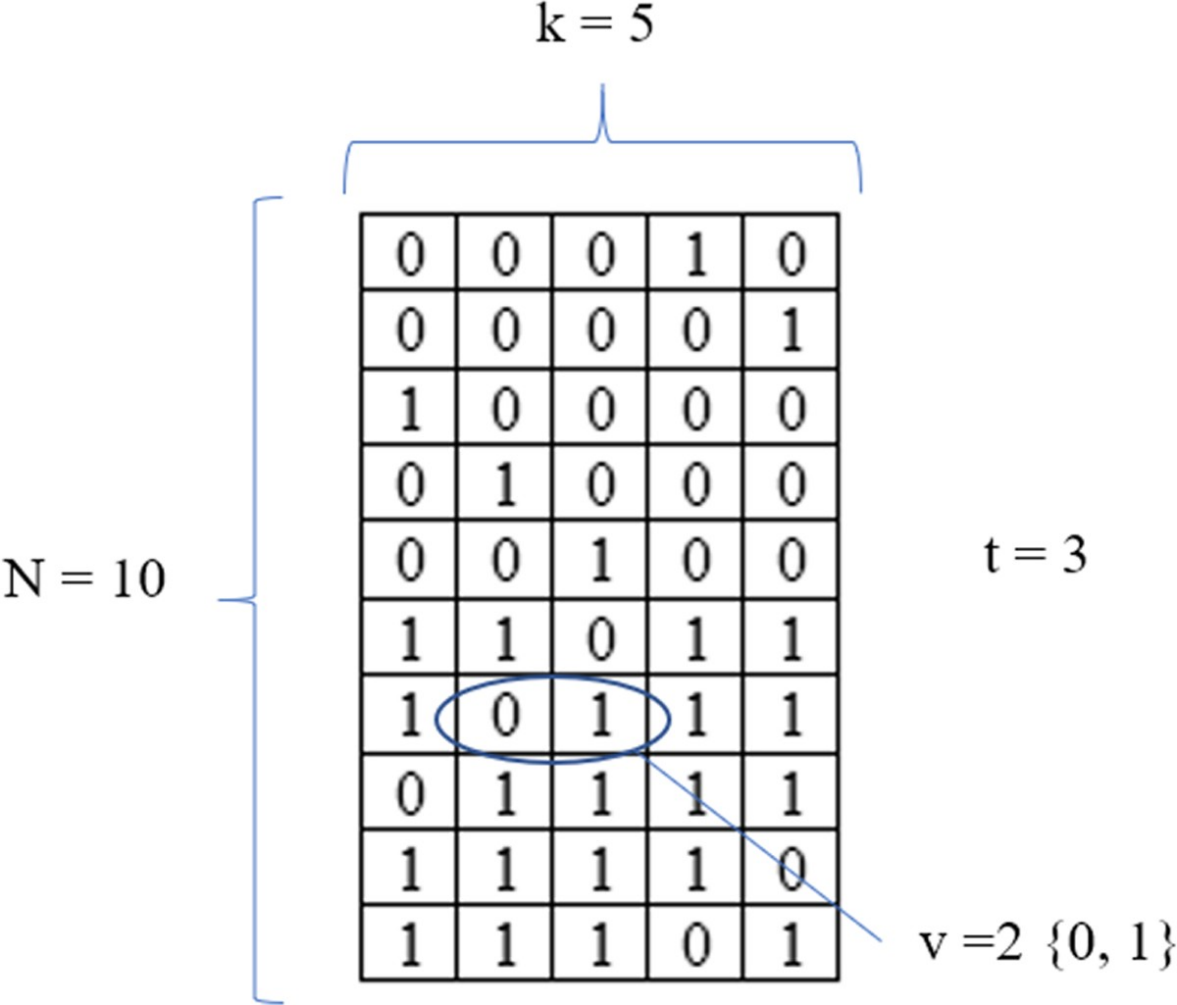
Example of an optimal CA (10; 3, 5, 2).

To get an insight into the use of a CA, assume that we have a software component with *5* variables (also known as parameters in software development), each one having *2* values and with strength *3*, an optimal CA has *10* rows (optimal means that the number of rows is a minimum). This CA is denoted with the notation *CA (N; t*, *k*, *v) = CA (10; 3*, *5*, *2)*. It can be seen in Fig 1 that each three columns it appears (at least once) each of the possible value combinations {*000*, *001*, *010*, *011*, *100*, *101*, *110*, *111*}. This CA can be used to test all *3*-way combinations of *5* binary variables.

**Fig 2:**
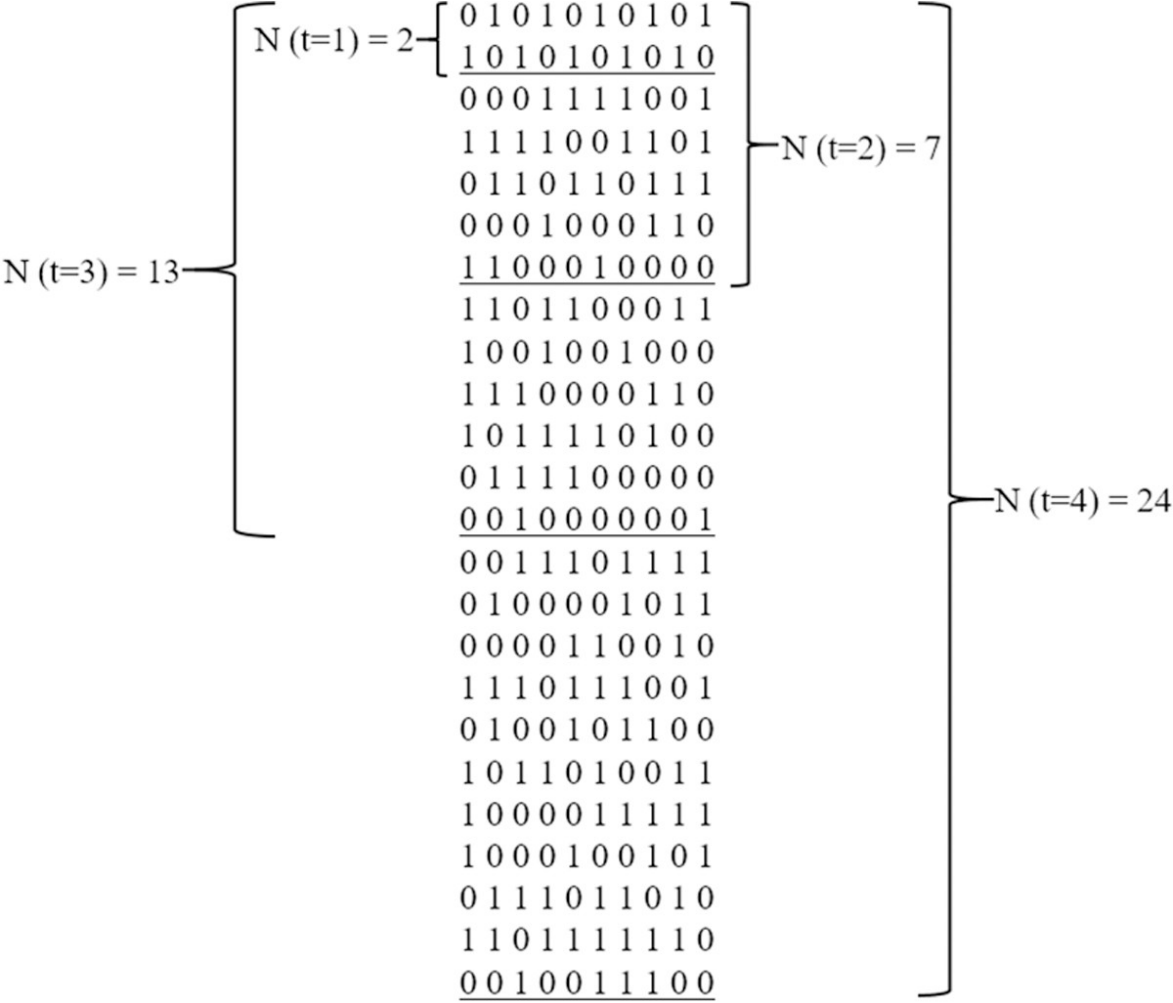
An example of an ICA (2, 7, 13, 24; 4, 10, 2).

Fig 2 shows an example of an ICA (*2*, *7*, *13*, *24; 4*, *10*, *2*). This ICA contains in its first two rows a CA (2; 1, 10, 2). In its first seven rows a CA (7; 2, 10, 2). In its first 13 rows a CA (13; 3, 10, 2). In its 24 rows a CA (24; 4, 10, 2).

## Proposed method

A clustering algorithm involves multiple tasks, such as the identification and modification of diverse parameters and processes. The results of the algorithm depend on the selection and pre-processing of the initial parameters. A good tuning process can increase the quality of the grouping regarding *cohesion* (how close or similar the elements are within a cluster) and separation (how far the created groups are from each other). In this paper, three main components are defined: ì) metric, or measure of similarity between business processes within the same cluster, ii) strength of the ICA needed to find the grouping with greatest intra-cluster and extra-cluster distribution, iii) number of groups needed to get an optimal grouping (ICA alphabet). The steps to select the parameters of the proposed method are described below.

### Identification of similarity metrics

Clustering optimization requires the accurate definition of closeness between a pair of objects, regarding either similarity or distance. The literature presents several similarity or distance measures, such as Euclidian distance and cosine similarity. Selection of the most appropriate similarity measure is crucial for the proper performance of the clustering algorithm. This closeness measure allows locating new data objects (in this case, business process) in the group with the highest level of similarity [47]. For the evaluation of similarity measures, the following must be considered [48]: 1) the distance between two BPs cannot be negative, d (BP_x_, BP_y_) ≥ 0; 2) the distance between two BPs must be zero if and only if two objects are identical, namely, d (BP_x_, BP_y_) = 0 if and only if BP_x_ = BP_y_; 3) the distance must be symmetric, i.e. the distance of BP_x_ to BP_y_ is the same distance as BP_y_ to BP_x_, d (BP_x_, BP_y_) = d (BP_y_, BP_x_); and 4) it must fulfil the triangle inequality, i.e. d (BP_x_, BP_z_) < = d (BP_x_, BP_y_) + d (BP_y_, BP_z_). The evaluated measures are described below bearing in mind the notation of Table 1.

**Table 1:** Notation for the tuning of the grouping.

Symbol	Description
d	Total number of dimensions (textual and structural) for each business process in the repository.
x	Business process represented as a vector with all components (d dimensions).
*C*_*i*_	The *i*^*th*^ cluster.
*c*_*i*_	Centroid of cluster *C*_*i*_ represented as a vector with d dimensions.
c	Average of all business processes represented as a vector with d dimensions.
m_i_	Number of business processes in the *i*^*th*^ cluster.
M	Number of business processes in the data set (result list). This value defines the ICA (k = M) used for grouping.
g	Number of clusters or groups.

#### Euclidean distance

This measures the distance between two BPs represented as vectors of terms containing textual and structural information. The Euclidean distance between a specific BP (x) and the centroid of the i^th^ cluster (c_i_) is defined by Eq 1. This equation is based on vectors with d dimensions.

$$D_{e}=\sqrt[{2}]{\sum_{j=1}^{d}{\left(c_{i,j}-x_{j}\right)}^{2}}$$ (1)

#### Cosine similarity

The similarity of two BPs is defined as the cosine of the angle between the vectors (each value in the vector is greater than or equal to zero) that represent the BPs. Thus, when the value of the cosine of the angle is close to 1, the evaluated BPs shows a high degree of similarity. Conversely, when the cosine value tends to 0, it means similarity is low. The cosine similarity between a specific BP (x) and the centroid of the i^th^ cluster (c_i_) is defined by Eq 2).

$$SIM_{c}=\sum_{j=1}^{d}\left(x_{j}\times c_{i,j}\right)/\left(\sqrt{\sum_{j=1}^{d}x_{j}^{2}}\times \sqrt{\sum_{j=1}^{d}c_{i,j}^{2}}\right)$$ (2)

#### Jaccard coefficient

This coefficient compares the weight of the sum of common elements (textual or structural) with the weight of the sum of terms in BPs that are not common (see Eq 3). Before to calculating this coefficient, each value greater than zero in the BP representation is assigned to one (1) to work in a binary space.

$$SIM_{j}=\sum_{j=1}^{d}\left(x_{j}\times c_{i,j}\right)/\left(\sum_{j=1}^{d}x_{j}^{2}+\sum_{j=1}^{d}c_{i,j}^{2}-\sum_{j=1}^{d}\left(x_{j}\times c_{i,j}\right)\right)$$ (3)

#### Manhattan distance

This is calculated as the difference between two vectors that represent two BPs in a vector space representation. The absolute difference is calculated based on the textual and structural components of the BPs (see Eq 4).

$$D_{m}=\sum_{j=1}^{d}\left|c_{i,j}-x_{j}\right|$$ (4)

### Clustering algorithm

**Fig 3:**
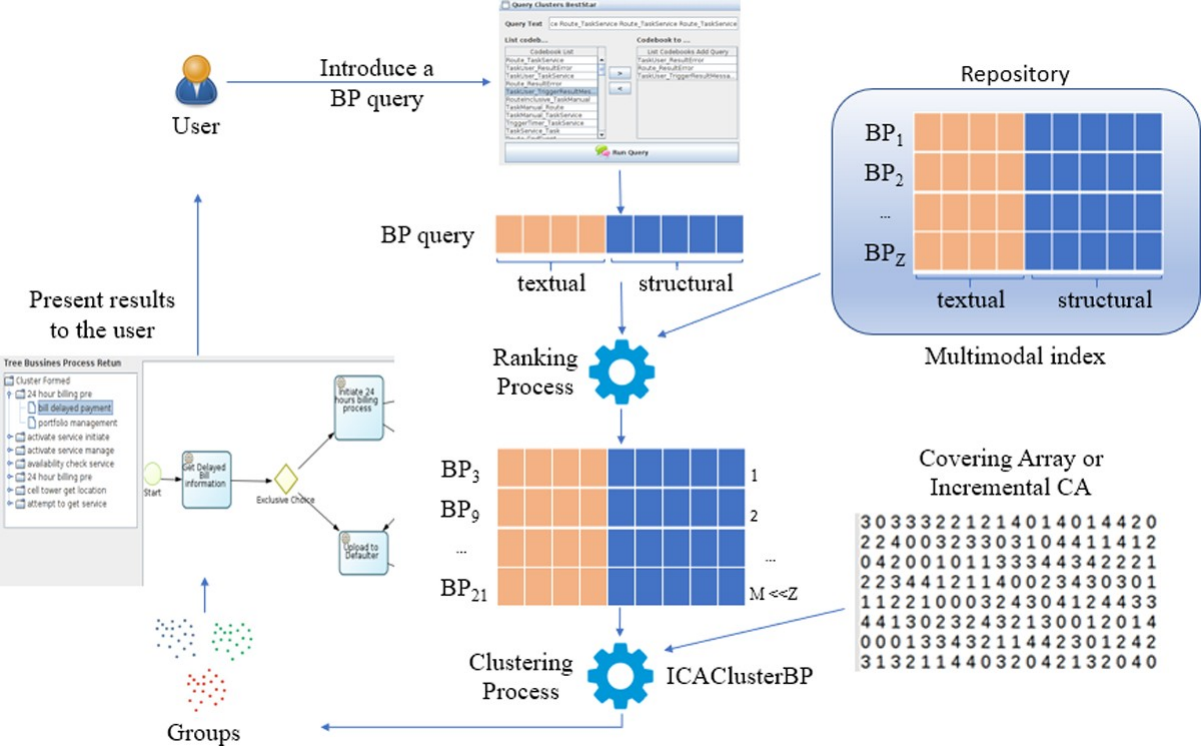
The entire process of query, search, and grouping.

The process (Fig 3) starts when the user defines a query Business Process using a Form (this query can be based on textual or structural information—or both). This query is transformed into its vector space representation (a detailed description can be found in (H. Ordoñez, Ordoñez, et al., 2016) and (Figueroa et al., 2016)). This query BP represented as a vector is compared with the BP stored in the repository (using the same representation). A ranking process generates a list of the BPs in the repository according to their similarity to the query BP. Using the BPs retrieved from the search, the clustering algorithm, ICAClusterBP, creates a set of groups. These groups are displayed in hierarchical folders. This method uses the textual and structural information of the BPs. Thus, groups are created based on the similarity (textual and structural) of the retrieved BPs. To sample the minimum number of possible clustering solutions, a list of ICAs is used. The alphabet of an ICA is used to determine the number of sampled groups. Likewise, the strength of an ICA indicates the number of BPs that will be sampled in all its possible ways of grouping.

The clustering algorithm consists of the following elements:

#### List of ICAs with different alphabets

The clustering algorithm uses a list of ICAs. A particular ICA has the alphabet fixed and as the processing of the rows of the ICA is done all the possible strengths are covered. This list of ICAs was created off-line and then stored in a database.

#### Grouping

This component uses the *ICAClusterBP* algorithm. The algorithm takes as input a list of ICAs with different alphabets (the *i*-th ICA of the list is denoted by ICA_*i*_ and its alphabet by *v*_*i*_). The algorithm in each query evaluates all the rows of each ICA_*i*_, and then selects the best grouping, i.e. the grouping with the lowest BBIC (calculated using Eq 9). This grouping allows identifying the optimal value of strength and the optimal value of the alphabet *v*_*i*_ for such query. For each query, each ICA_*i*_ contains a sample of the possible solution. The algorithm takes each row of the ICA_i_ to form a clustering solution. Once each Business Process is assigned to its respective group the quality of that solution is evaluated according to the value of the intracluster *Sum of Squared Error (SSE*). The algorithm repeats the process with all the rows of an ICA until the best grouping is found, according to the lowest value of *BBIC*. The algorithm uses the parameters previously presented in Table 1. The steps of the *ICAClusterBP* algorithm are described below.

**Step 1**: the algorithm takes each row of the ICA_i_, which represents a possible grouping and forms the groups using each column of a row.
**Step 2**: Once the groups are formed, the algorithm calculates the centroid of each group using Eq 5 over each dimension of the centroid vector, therefore, j = {1, …, d}.

$$c_{i,j}=\frac{1}{m_{i}}\sum_{x\in C_{i}}x_{j}$$ (5)

**Step 3**: Once *c*_*i*_ is calculated, *SSE*_*i*_ is calculated for each cluster *C*_*i*_, using Eq 6.

$$SSE_{i}=\sum_{x\in C_{i}}distance{\left(c_{i},x\right)}^{2}$$ (6)

*distance* is calculated using the equation with the best results during the evaluation process. As will be explained in the next section, the best results were obtained with cosine similarity using vector space representation of BPs, including textual and structural information. In this case, *distance = 1 –cosine similarity*.

**Step 4**: SSE is calculated for the whole grouping solution using Eq 7.

$$SSE=\sum_{i=1}^{g}SSE_{i}$$ (7)

**Step 5**: Calculate Average Distance Between Centroids (ADBC) and Balanced Bayesian Information Criterion (BBIC) values for the whole grouping solution using Eqs 8 and 9.

$$ADBC=\frac{2}{g*\left(g-1\right)}\sum_{j=1}^{g-1}\sum_{l=j+1}^{g}distance\left(c_{l},c_{j}\right)$$ (8)

$$BBIC=M*Ln\left(\frac{SSE}{M*ADBC}\right)+g*Ln\left(M\right)$$ (9)

In Eq 9, SSE is the value obtained in Eq 7.

**Step 6**: Repeat steps 1 to 5 for all the rows of each ICA, and then return the best grouping (group with lowest BBIC). The process can also be stopped when the maximum execution time is reached; similarly, if a quality level is reached, based on the BBIC value of the best-found solution.
**Step 7 or Clusters display**: This component displays the created groups in an organized and categorized way. This structure enables users to review and select the groups with higher similarity to the query.

Clustering algorithms normally report local optimal solutions, but given the flexibility and simplicity of the proposed method, it is feasible to include *k-means* or *Expectation-Maximization clustering using Gaussian Mixture Models* (*EM-GMM*), among others, for improving the best solution found (the solution can be taken as the initial solution of the *k-means* or *EM-GMM* algorithms) with the purpose of regrouping the BPs if necessary. It is known that the results obtained from these algorithms depend on the initial solution. In this case the results of the proposed method define the best place in the search space for the local optimization to be executed. Also, this approach can be applied to any row in the ICA between Steps 1 and 2, and so each row of the ICA can be viewed as the initial solution for the local clustering algorithm and all of these solutions compared using BBIC to select the best one. These additional proposals are not analyzed in this paper; consequently, they are defined as future work.

### Example of execution of ICAClusterBP algorithm

**Fig 4:**
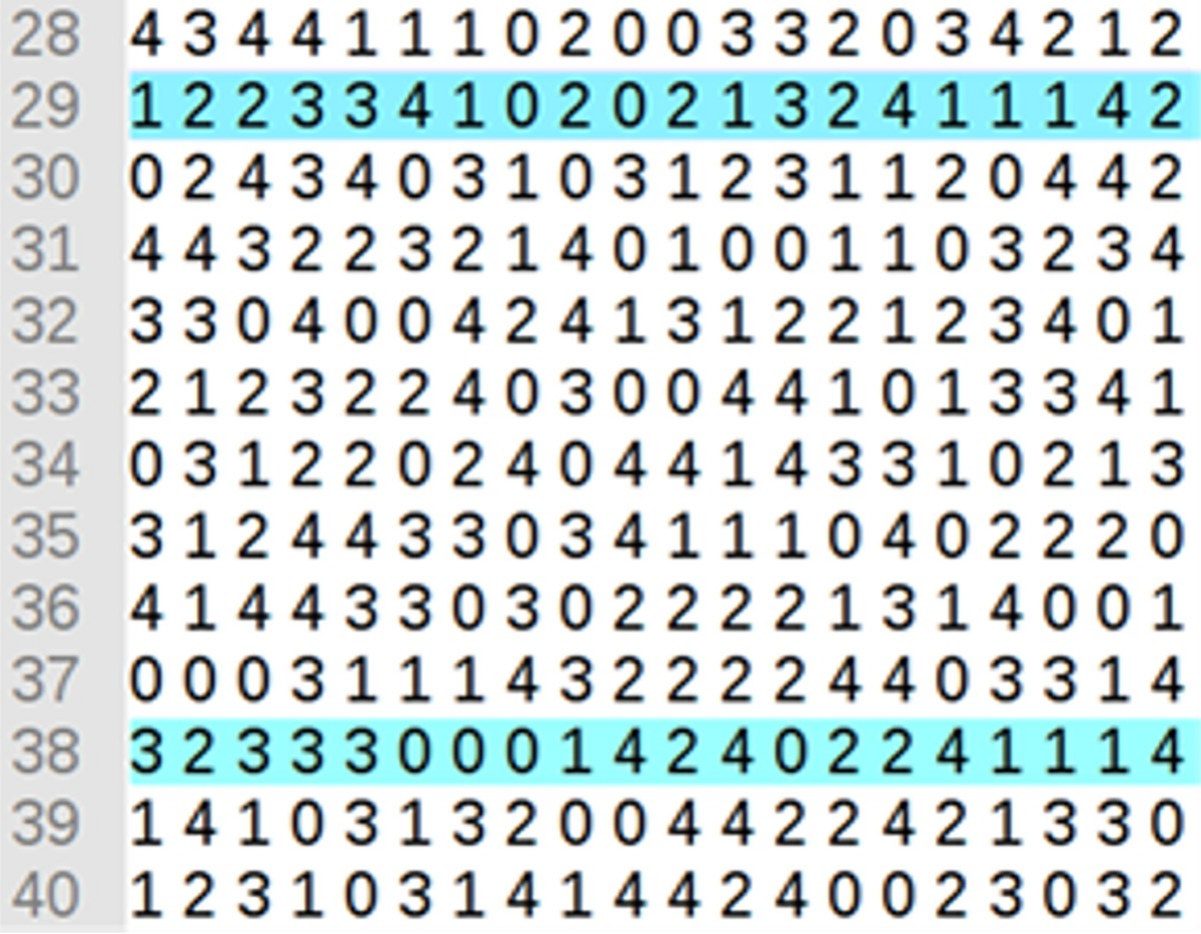
Part of the ICA (5, 40; 2, 20, 5) used for grouping.

To illustrate the clustering process, we will use ICA (5, 40; 2, 20, 5), i.e. 20 BPs using 5 clusters at most and testing at least once for each two BPs that appear as belonging to the groups: {{0,0}, {0,1}, …, {0,4}, {1,0}, …, {4,4}}. Part of ICA (5, 40; 2, 20, 5) is shown in Fig 4.

**Fig 5:**
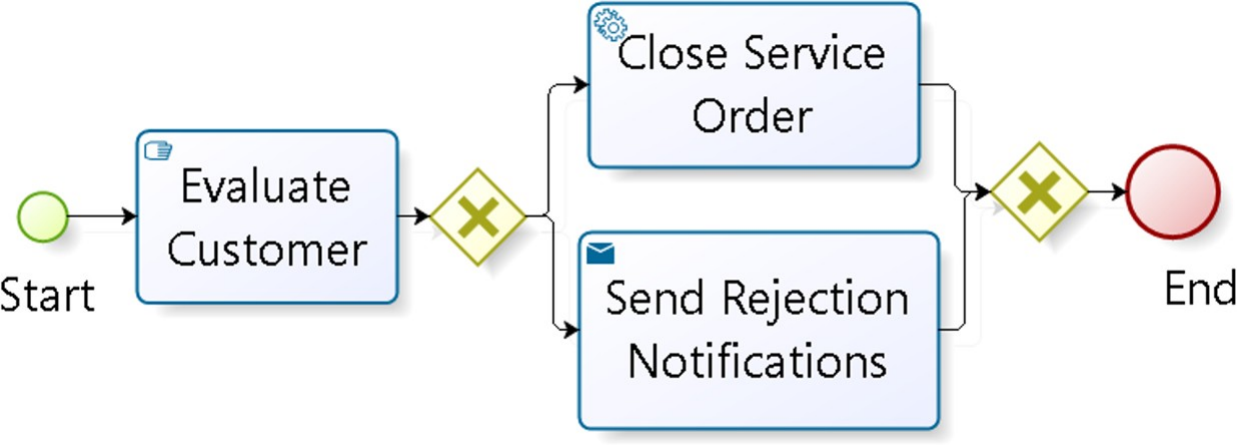
Query BP.

**Fig 6:**
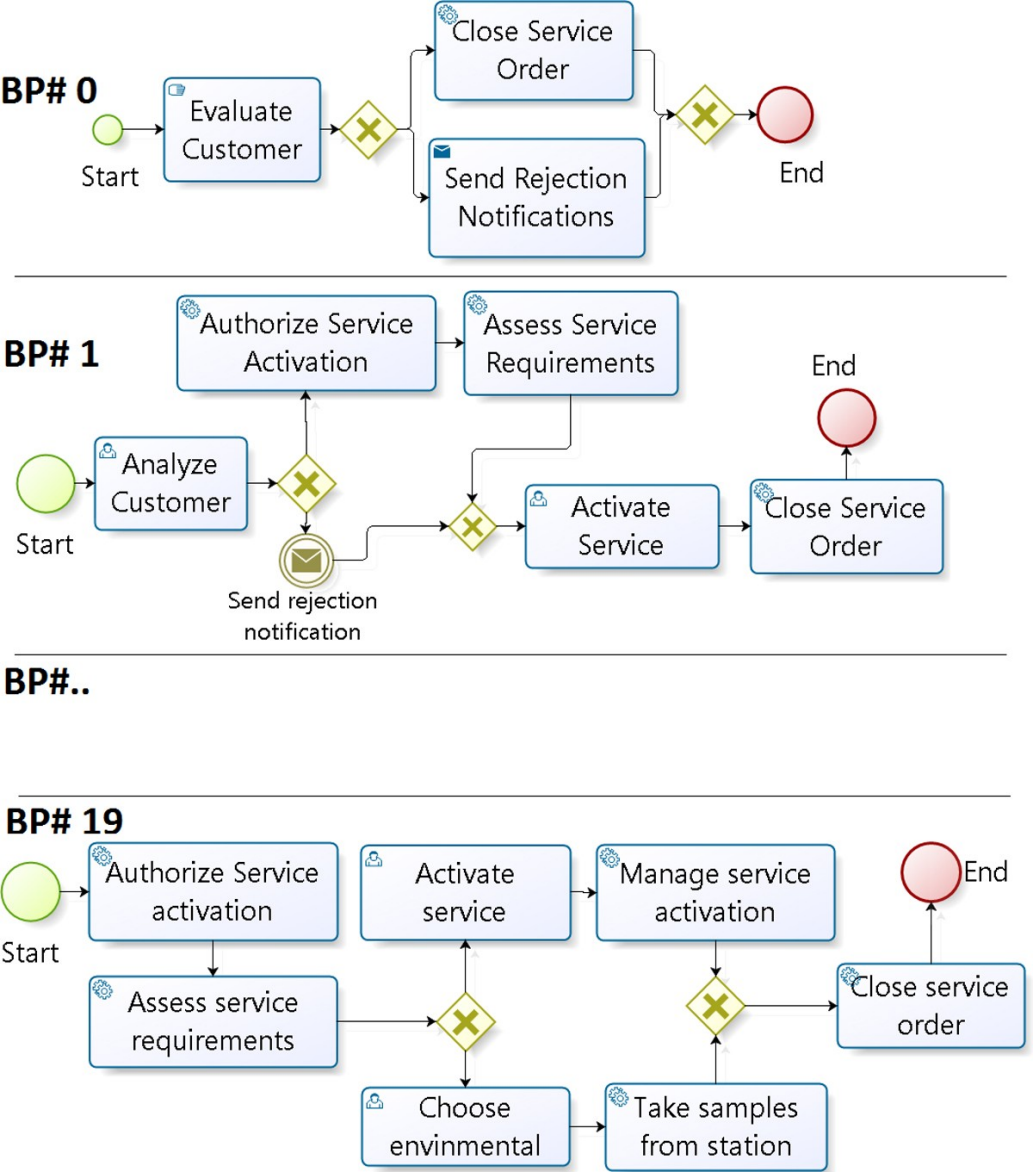
List of results.

Fig 5 shows the "Activate services" business process used as a query BP. The multimodal search retrieves the BPs with the greatest similarity to the user query and generates a list of results filtered and sorted in descending order (Fig 6).

**Fig 7:**
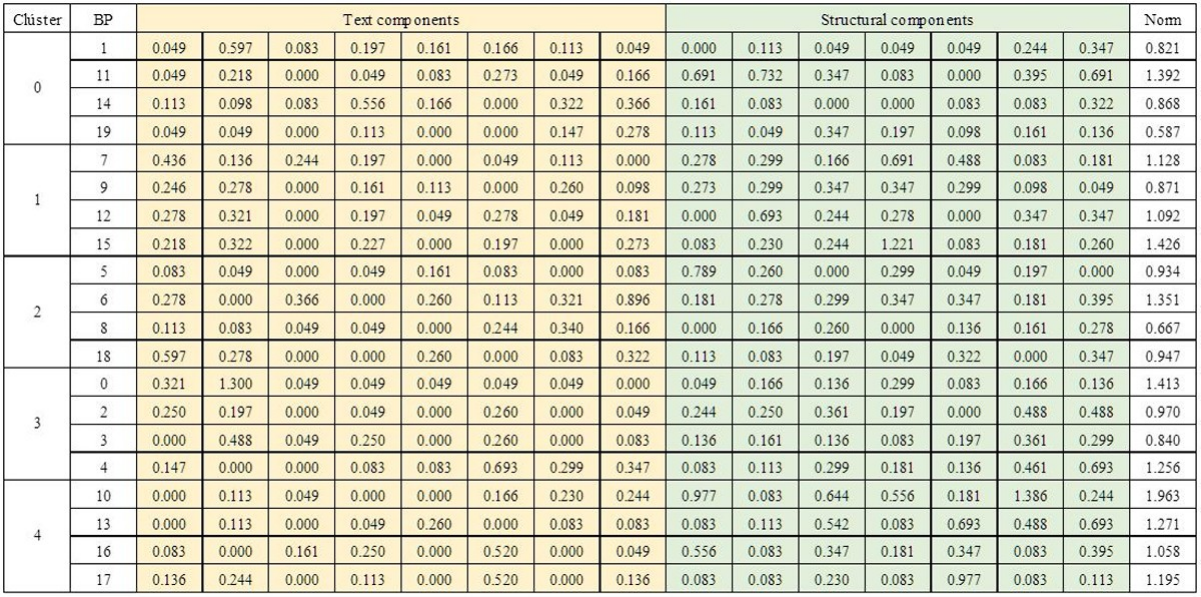
BPs organized by cluster using the ICA.

The *ICAClusterBP* algorithm then takes each one of the rows of the ICA (Fig 4) to group the results using the following steps:

**Step 1**: the first step is to create the clusters, taking each row of the ICA. The name of each group corresponds to a value of the ICA alphabet (this may be 0 to 4). The index value of each column is equal to the index value in the result list. Thus, the BP in column 0 corresponds to the BP retrieved as item 0 (the first in the result list). As an example, the groups created with the first row in Fig 4 (row 18 = {3 0 3 3 3 2 2 1 2 1 4 0 1 4 0 1 4 4 2 0}) are (see Fig 7 with a sample of weights in the vector representation of each BP including textual and structural data. This figure also shows the norm of each vector): **Cluster 0** (C_0_) composed by BP1, BP11, BP14, and BP19, **Cluster 1** (C_1_) composed by BP7, BP9, BP12, and BP15, **Cluster 2** (C_2_) composed by BP5, BP6, BP8, and BP18, **Cluster 3** (C_3_) composed by BP0, BP2, BP3, and BP4, and finally, **Cluster 4** C_4_) composed by BP10, BP13, BP16, and BP17.

**Fig 8:**
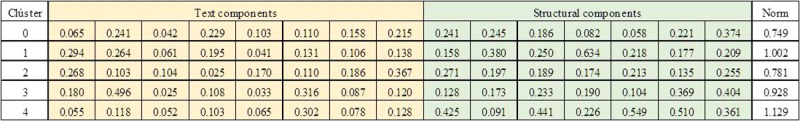
Centroids of each cluster.

**Step 2**: to calculate the centroid of each group using Eq 5. All centroids (c_i_) are calculated and stored. These centroids have the same representation of the business process (real vector containing the average of textual and structural information of the business processes in each cluster) as can be seen in Fig 8.

**Fig 9:**
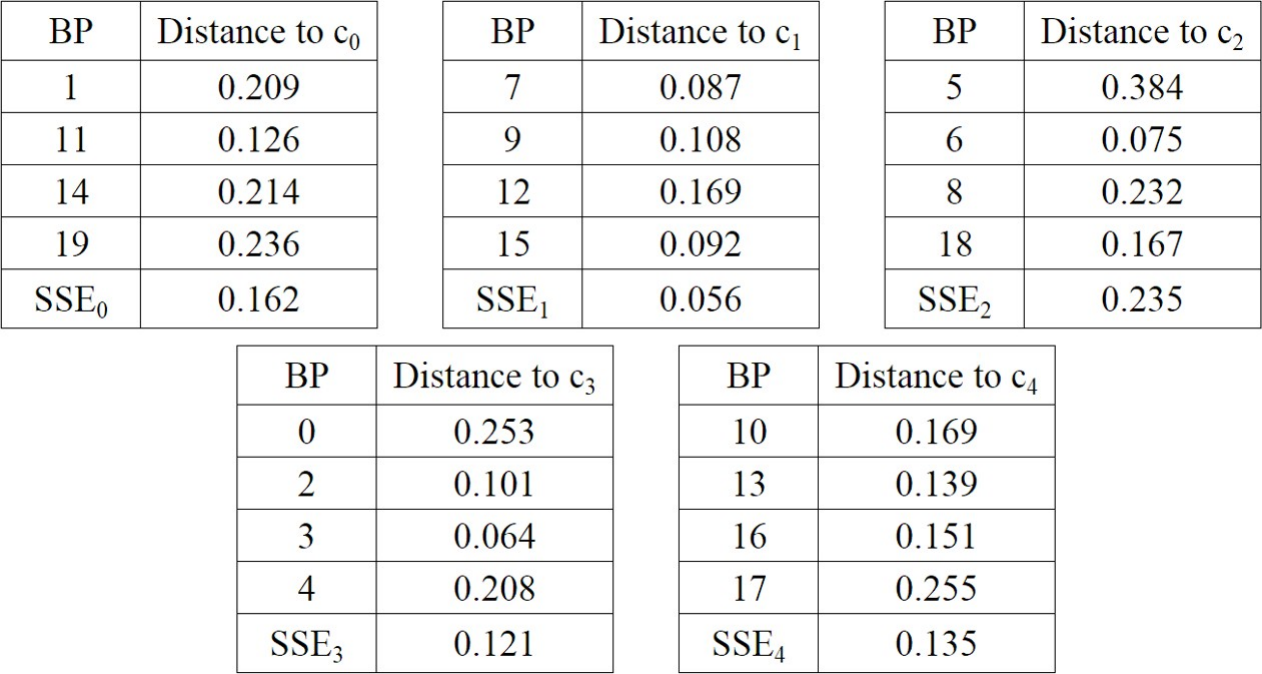
SSE values for each cluster.

**Step 3**: to calculate SSE_i_, for each cluster *C*_*i*_ using Eq 6. For the present example, the value of distance (1 –cosine similarity) in Eq 6 was calculated (see Fig 9).

**Fig 10:**
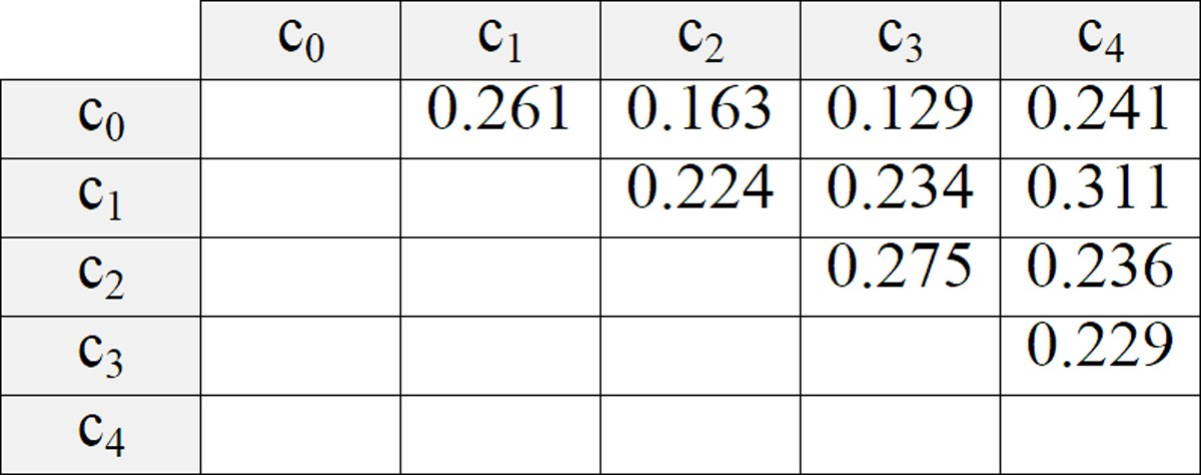
Distance between centroids to calculate the ADBC value.

**Step 4**: to calculate the total intra-cluster SSE using Eq 7, for this example, SSE = 0.710.
**Step 5**: to calculate the total ADBC = 0.230 and BBIC = -22.429 using Eqs 8 and 9. To obtain the ADBC value, it is necessary to calculate each pair of distances between centroids as in Fig 10.

**Step 6**: to repeat steps 1 to 5 until all the groupings recorded in the ICA are covered.
**Step 7**: in this step, the groups in the lowest BBIC grouping are shown to the user.

**Fig 11:**
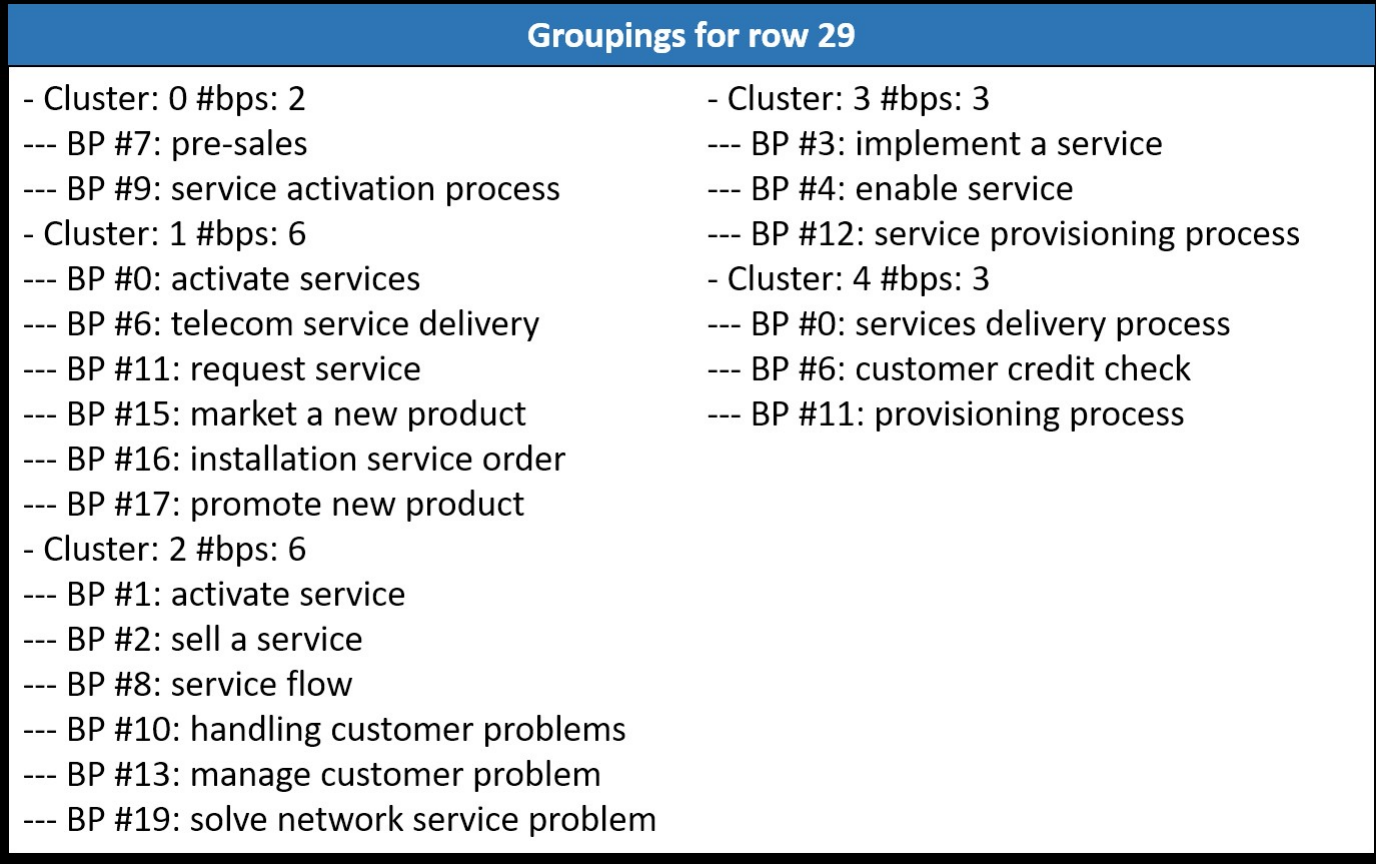
Groupings created using ICAClusterBP.

Finally, the algorithm returns the grouping with the lowest value of BBIC. In this example, the best solution corresponds to row 38 of the ICA (see Fig 4). Row 29 also obtains a good solution. Fig 11 shows a comparison of the grouping solution obtained by row 38 (the best) and row 29 (the second-best solution).

### Evaluation of the similarity measures

For the evaluation of the similarity measures, the same BPs and queries of [8] were used. This process was conducted to define the similarity measure that will be utilized later in the algorithm.

Table 2 shows the values of the SSE evaluation, where cosine similarity obtained the lowest values of SSE. This result is consistent with previous research, where this measure showed the best results when the BPs elements (e.g., text, structure, or both) use vector space representation. Considering the above, the following evaluations are made taking the cosine similarity measure for the proposed algorithm.

**Table 2:** Results of evaluation of similarity measures (best results in bold).

SSE evaluation							
Measure	Q1	Q2	Q3	Q4	Q5	Q6	Average
**Euclidean distance**	0.39	0.298	0.182	0.29	0.419	0.471	0.342
**Cosine similarity**	**0.223**	**0.172**	**0.121**	**0.176**	**0.405**	**0.495**	**0.265**
**Manhattan distance**	0.583	0.387	0.219	0.478	0.702	0.51	0.480
**Jaccard coefficient**	0.813	0.845	0.501	0.886	0.567	0.704	0.719

### Identification of level of strength of ICA

This phase aims to determine the appropriate value of the strength of the ICA that obtains the grouping with the lowest BBIC. To achieve this, the *ICAClusterBP* algorithm was run with the following configuration: ICA (8, 97, 501, 2501; 5, 20, 3). The evaluations were carried out as follows: with strength 1, the first 8 rows of the ICA were evaluated; with strength 2, the first 97 rows were evaluated; with strength 3, the first 501 rows were evaluated; and with strength 4, 2501 rows were evaluated.

Table 3 shows that as the strength increases, the value of the intra-cluster *SSE* decreases until reaching 0.507 with strength 4. This result suggests that as the number of evaluated groupings (number of rows in an ICA) increases, the created groups become more cohesive–i.e. the created groups are closer and contain greater similarity between their elements.

**Table 3:** Evaluation of level of strength t (best results in bold).

Evaluation of strength *t*							
level of *t*	Q1	Q2	Q3	Q4	Q5	Q6	Average
**t = 1**	1.432	1.020	1.350	0.660	1.740	1.670	1.312
**t = 2**	0.520	0.328	0.210	0.520	1.630	1.120	0.721
**t = 3**	0.457	0.306	0.189	0.341	1.270	0.860	0.571
**t = 4**	**0.421**	**0.295**	**0.135**	**0.321**	**1.090**	**0.780**	**0.507**

Moreover, the results in the queries evidence that as strength increases the value of *SSE* decreases significantly, as in the case of Q1 where *SSE* decreases 240% with strength t = 1 compared to strength t = 4. Equally, in Q3 from strength 1 to strength 4, *SSE* decreases 900%, and in general, from strength 1 to strength 4, *SSE* decreases by 159%. These values corroborate that as strength increases, the possibility of the algorithm finding a better grouping increases.

Based on the results achieved in the evaluation of the level of strength t, it can be inferred that strength t = 4 will be used to perform the evaluation process of the accuracy and completeness of the *ICAClusterBP* algorithm.

### Evaluation of number of groups (parameter v of an ICA) to get the best grouping

The upper bound on the number of groups is defined by the alphabet of the ICA. This parameter affects the performance of the algorithm, since the computational time of the algorithm is proportional to the number of groups. Besides, accuracy is also affected by the groups that exist in the ideal grouping. Furthermore, generating solutions with a small upper bound on the number of groups caused the grouping process to leave out elements (BPs) that may be relevant to a group, thereby decreasing the similarity between elements in each group as well as the accuracy of the overall grouping. As a result, determining an optimal number of groups to form can improve the quality of the grouping regarding the similarity of the elements in each group and the separation between groups.

Based on the above, the number of groups depends not only on the number of elements (BPs) but also depends on the number of features of these elements (BPs). The number of groups is based on the combination of features as well as the number of elements. Therefore, the features and elements are crucial during the clustering process.

In the present work, BBIC [14] was used to determine the ideal value of the upper bound on the number of groups to be created. For this purpose, the algorithm was run using an ICA with alphabets *v = 2* to *v = 6* (ICA (3, 12, 28, 39; 4, 20, 2), ICA (6, 29, 121, 314; 4, 20, 3), ICA (4, 54, 273, 760; 4, 20, 4), ICA (10, 73, 373, 1865; 4, 30, 5), and ICA (14, 128, 1095, 4820; 4; 20, 6). Available at: http://www.tamps.cinvestav.mx/~oc/CLUSTERING).

In this process, the algorithm carries out each query in each ICA with the aim of finding the lowest value of BBIC. When this value is found, the value of alphabet v is taken as a reference to create the grouping. The BBIC is calculated using Eq 9.

**Fig 12:**
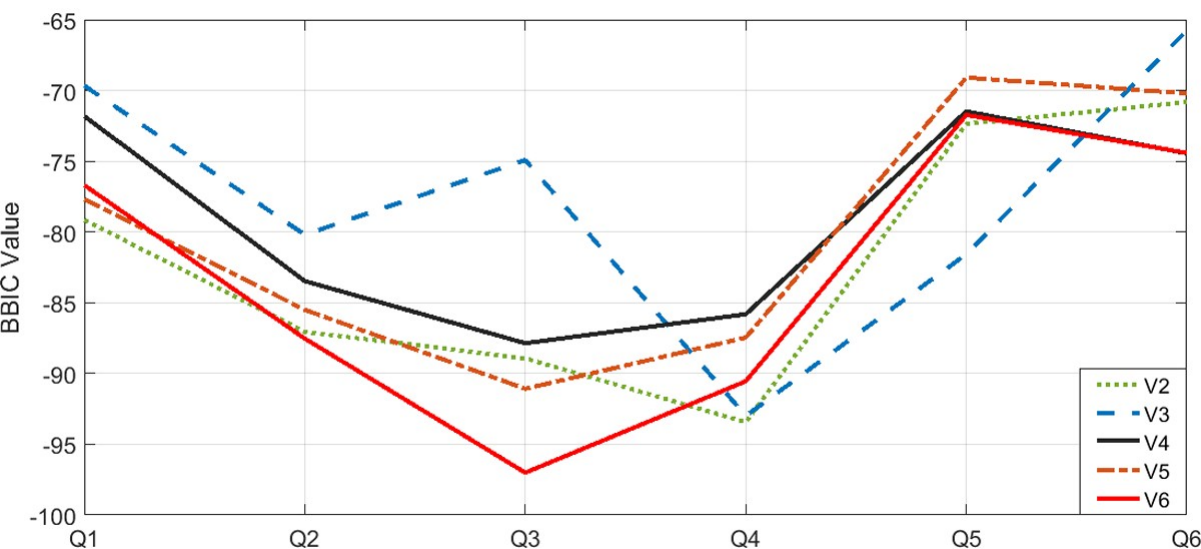
Value of BBIC in each one of the queries for each alphabet in each ICA.

Fig 12 shows the results obtained while finding the ideal number of groups to be created using the BBIC. This figure indicates that the ICA with strength 4 and alphabet v = 6 obtains the lowest value of BBIC for queries Q2, Q3, and Q6. In Q1 and Q4, alphabet v = 2 obtains the best results and alphabet v = 3 obtains the best results in Q5. In Q2 and Q5, alphabet v = 2 obtains the second minimum value of BBIC. In Q1 and Q3, this second place is obtained by alphabet v = 5. Although alphabet v = 3 obtains the worst results in Q1, Q2, Q3 and Q6, it obtains the best result in Q5 and the second best in Q4. These results reveal that the number of groups is not directly related to the alphabet, but high alphabets allow exploring a greater number of possible groups. Conversely, the number of groups is defined by the similarity of the BPs retrieved during the search phase (algorithm input).

## Evaluation and results

Measuring grouping quality or clustering performance is not a trivial task because there are no standard methodologies for this purpose. Consequently, clustering evaluation is based on several metrics that evaluate the internal and external quality of created groups [49]. Internal evaluation relies on the fact that groups that are compact and well separated from other groups are better. The present evaluation measures: density, distances between business processes in the same group (the smaller the distance, the more compact the groups), and separation between clusters (the larger the distance, the higher–and better—the separation between groups) [50].

External evaluation is used, meanwhile, when an "ideal" training data set exists, the class or classes of business process are known, and they are compared with the groups created by the evaluated algorithm. Consequently, external validation is more accurate than internal. The latter is important when it is necessary to find the best clustering method for a particular task and usually involves comparing a variety of algorithms on specific datasets [51].

The evaluation was conducted in three phases: internal evaluation, external evaluation, and analysis of statistical significance. Results obtained using the present tuning model were compared with the results of a manual evaluation performed on a closed test set described in [15]. This set was collaboratively created by 59 experts. Furthermore, the results of the *ICAClusterBP* algorithm were compared against the results of four state-of-the-art algorithms; *CAClusterBP* presented in [16] which used an ICA (5, 40; 2, 20, 5) and Euclidean measure to define the similarity of the BPs within each group; the HC algorithm [32], which uses cosine coefficient to measure the similarity between two process models and implements a hierarchical agglomerative algorithm for clustering; *k-meansBP* that uses the *k-means* clustering algorithm (adapted for use in BPs) and a multimodal search model based on cosine distance [41]; and finally, *GroupBPFuzzy* presented in [8]—this algorithm uses a multimodal method to retrieve BPs from a repository and an incremental algorithm to group retrieved BPs. Groups are created using a fuzzy-based function that measures similarity between two BPs according to the number of common elements between them.

### Internal evaluation

The first evaluation does not require human intervention and uses internal metrics. These metrics are used to identify how close or distant the BPs are from each other in the groups formed. Two repositories were used for this evaluation: Repository 1 is Descript [15] and Repository 2 is a subset of Apromore [5] with 142 BPs described using BPMN. The latter repository is used only in the internal evaluation since the predefined set of queries and the ideal results are not known. The metrics are described below.

#### Sum-of-squares between (SSB)

This measures the separation between clusters (high values are desired). In Eq 10, *g* is the number of clusters, *m*_*i*_ is the number of elements in the cluster *i*, *c*_*i*_ is the centroid of cluster *i, and $\overset{-}{x}$* is the mean of the data set [52].

$$SSB={\sum_{i=1}^{g}m_{i}\;distance{\left(c_{i}-\bar{x}\right)}^{2}}_{}^{}$$ (10)

#### Sum-of-squares within (SSW)

This measures variance (low values are desired) within groups, based on each of the existing elements in each group [52]. This measure is expressed by Eq 11 where *g* is the number of clusters, *x is* a bussiness process in cluster *C*_*i*_ and *c*_*i*_ is the centroid of cluster C_*i*_. This metric is also know as SSE.

$$SSW=\sum_{i=1}^{g}\sum_{x\in c_{i}}\;distance{\left(c_{i},x\right)}^{2}$$ (11)

#### Davis building index (DB)

This measures the relation of the dispersion within the cluster and the separation between clusters. Low values are desirable. This measure is used to evaluate the formation of unique groups [53] and is expressed by Eq 12 where *g* is the number of clusters, *σ*_*i*_ is the average distance between each BP in cluster *i* and the centroid, *σ*_*j*_ is the average distance between each BP in cluster *j* and the cluster centroid. Finally, $distance(c_{i},c_{j})$ is the distance between the centroids of clusters i and j.

$$DB=\frac{1}{g}\sum_{i=1,i\neq j}^{g}max\left(\frac{\sigma_{i}+\sigma_{j}}{distance\left(c_{i},c_{j}\right)}\right)$$ (12)

Table 4 shows the results obtained during the internal evaluation. Regarding SSB, maximum values 0.619 and 0.627 are achieved using *ICAClusterBP*. This value indicates that the grouping created using ICA contains groups with high separation, i.e., the elements (BPs) within a group *i* are highly different from those of a group *j*. Also, the elements (BPs) belong only to a single group, thus avoiding overlapping and shared elements between groups. For its part, *ICAClusterBP* outperforms *HC* by 332–384%; this is because *HC* uses a hierarchical algorithm that does not allow dynamic assignment or reallocation of BPs. *ICAClusterBP* outperforms *k-meansBP* by 134–147% and *GroupBPFuzzy* by 81–88%; this is because *GroupBPFuzzy* creates groups of BPs without considering the minimal precision threshold, thus the distance between elements in each group increases. Finally, *ICAClusterBP* outperforms *CAClusterBP* by 60–63%; this result is due to the fact that *CAClusterBP* uses a CA with strength 2, and therefore it compares the combinations of at least pairs per group. Conversely, *ICAClusterBP* (ICA with strength 4) compares the existence of combinations of four elements per group.

**Table 4:** Results of internal evaluation (best results in bold).

Algorithm/measure	SSB		SSW		DB	
	Repository 1	Repository 2	Repository 1	Repository 2	Repository 1	Repository 2
***HC***	0.128	0.145	0.080	0.076	0.572	0.502
***k-meansBP***	0.251	0.268	0.048	0.043	0.583	0.493
***CAClusterBP***	0.380	0.392	0.039	0.036	0.137	0.123
***GroupBPFuzzy***	0.330	0.347	0.040	0.037	0.167	0.151
***ICAClusterBP***	**0.619**	**0.627**	**0.021**	**0.018**	**0.113**	**0.102**

Regarding SSW, variation of the elements between created groups using ICAClusterBP is low, unlike HC where the existing elements in each group must share textual and structural information in the group with higher similarity to them. *ICAClusterBP* outperforms *HC* by 74–76%. This result was achieved because *HC* creates a hierarchical structure using a dendrogram in which some groups exist inside other groups. For this reason, there are elements repeated across diverse groups. *ICAClusterBP* meanwhile outperforms *k-meansBP*, *CAClusterBP*, and *GroupBPFuzzy* by 56–58%, 46–50%, and 48–51% respectively. These results are due to the fact that *ICAClusterBP* uses strength 4, evaluating the interaction of four different elements at least once in the process of searching for the best grouping. Thus, the elements (BPs) belonging to each group present high similarity.

Moreover, *DB* evidences that elements are well placed within each cluster. In other words, the elements within a group are not dispersed (according to the features they have in common). Results of the internal evaluation vary because Repository 2 contains nearly to 40 BPs models more than Repository 1. Notwithstanding the above, the values of the applied metrics show that the best results are obtained by *ICAClusterBP*.

### External evaluation

In this phase, the grouping created automatically was compared with the ideal grouping created by experts [15]. Metrics for external evaluation were: weighted precision, weighted recall, and weighted F-measure (a measure of the harmony between precision and recall). These measures have been used traditionally in information retrieval research [54].

To evaluate these metrics, the grouping created automatically using *ICAClusterBP* {*C*_*1*_, *C*_*2*_…‥*C*_*k*_} is compared with the ideal grouping $C_{1}^{i},C_{2}^{i},{\ldots .C}_{h}^{i}$ created by experts [15]. The evaluation included the following steps: (a) find for each ideal group, $C_{n}^{i}$ the distinct group $C_{m}$ most similar to the group being assessed (groups created using *ICAClusterBP*) and calculate $P\left(C,C^{i}\right)$ using Eq 13, $R\left(C,C^{i}\right)$using Eq 14, and $F(C,C^{i})$, using Eq 15. (b) Calculate weighted precision using Eq 16, weighted recall using Eq 17 and weighted F-measure using Eq 18.

$$P\left(C,C^{i}\right)=\frac{\left|C\cap C^{i}\right|}{\left|C\right|}$$ (13)

$$R\left(C,C^{i}\right)=\frac{\left|C\cap C^{i}\right|}{\left|C^{i}\right|}$$ (14)

$$F\left(C,C^{i}\right)=\frac{2P\left(C,C^{i}\right)R\left(C,C^{i}\right)}{P\left(C,C^{i}\right)+R\left(C,C^{i}\right)}$$ (15)

$$p=\frac{1}{T}\sum_{j=1}^{h}\left|C_{j}^{i}\right|p\left(C_{m},C_{j}^{i}\right)$$ (16)

$$R=\frac{1}{T}\sum_{j=1}^{h}\left|C_{j}^{i}\right|R\left(C_{m},C_{j}^{i}\right)$$ (17)

$$F=\frac{2PR}{P+R};\;T=\sum_{j=1}^{h}\left|C_{j}^{i}\right|$$ (18)

where *C* is a group of BPs and *C*^*i*^ is an ideal group of BPs.

**Fig 13:**
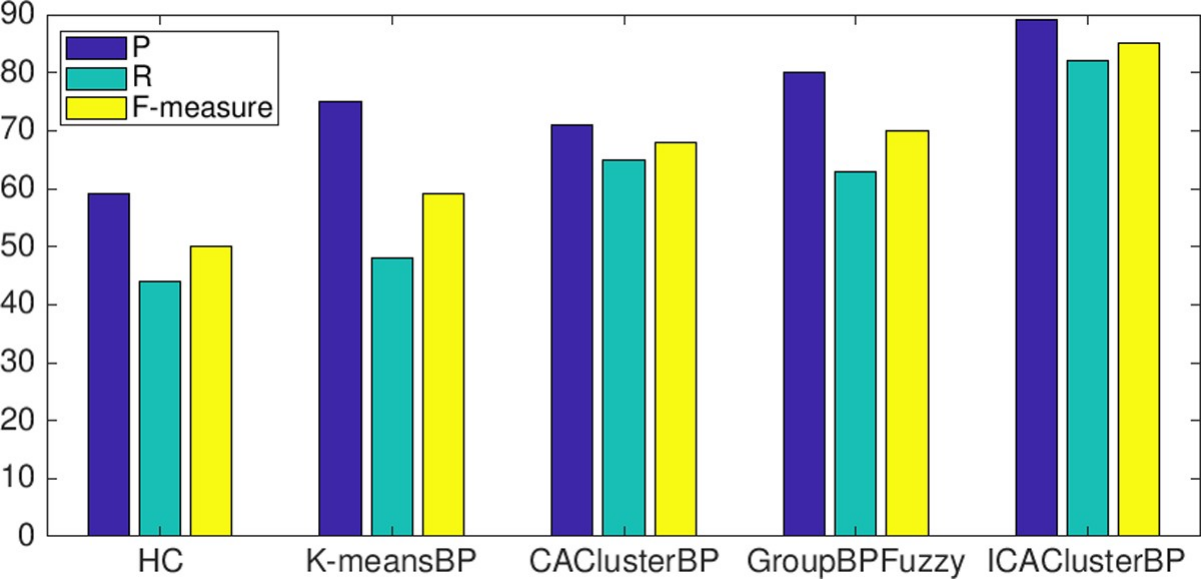
Values of precision, recall, and F-measure in external assessment.

Fig 13 shows the results of this phase. *ICAClusterBP* with an ICA reaches 89.7% precision (Rigor), exceeding *CAClusterBP* by 26%. This result is achieved because the groupings represented in each record of the ICA are more representative of the universe of possible groupings that can be generated with the search results (20). Thus, by increasing strength t from 2 to 4, it is possible to cover more possible distributions of BPs in groups (interaction) that have at least quartets, i.e. for every quartet of BPs all distributions are tested, in such a way that they belong to the same group at least once. *ICAClusterBP* exceeds the precision of *k-meansBP* by 21%. *HC* meanwhile obtains 52% less precision than *ICAClusterBP*; this may result because HC creates a dendrogram of BPs using exclusively structural information. Additionally, the creation of groups is done by statistically comparing BPs and the process allows a group to combine one group with a not quite similar BP (or two not quite similar groups) without the possibility to revert that grouping, and consequently grouping the precision decreases. The results of the precision evidence that the groupings created using *ICAClusterBP* achieve significant similarity with the human grouping. *GroupBPFuzzy* meanwhile achieved 12% less precision that *ICAClusterBP*. This is because *GroupBPFuzzy* starts allocating the BPs to groups without considering the similarity between the assigned BPs and the centroid of the group. Therefore, when a new business process is assigned to a group, the element with the lowest similarity is removed from the group and replaced with the new BP. As a result, some relevant elements remain unallocated, and therefore the number of false negatives increases and precision decreases.

Regarding recall, *ICAClusterBP* with 82.1% outperforms *CAClusterBP* by 26%. This value evidences that *ICAClusterBP* with strength t = 4 reduces the number of false negatives (FN) because each group contains at least quartets of BPs. Equally, the value of true positives (TP) (BPs located in the same group that was created manually is higher). *ICAClusterBP* meanwhile surpasses *k-meansBP* by 71%. The reason may be that during the relocation process this algorithm decreases the BPs located in the same group that was created manually. Finally, *ICAClusterBP* outperforms *GroupBPFuzzy* by 29%. In this case, the number of true positives (TP) increases along with the value and strength t, and hence at least four BPs are in the same groups of the manual grouping. On the other hand, the minimum value of BBIC makes it possible to achieve several groups with higher coherence with the number of elements of the group. Due to the above, the *ICAClusterBP* grouping has a high level of similarity with the grouping created by experts.

Regarding F-measure, *ICAClusterBP* (85.7%) achieves 31% better than *CAClusterBP*, 49% better than *k-meansBP*, 73% better than *HC* and 21% better than *GroupBPFuzzy*. This last fact makes it possible to infer that the grouping created with *ICAClusterBP* has greater harmony between precision and recall. For this reason, the groups created are most relevant and most like the groups created manually by experts.

### Analysis of statistical significance

Friedman (average ranking) and Wilcoxon (signed-rank) non-parametric statistical tests were used to evaluate statistical significance of the results shown in Fig 13. The Friedman test generated a ranking in ascending order according to the performance average, based on a chi-square distribution with 4 degrees of freedom. Table 5 shows the ranking results obtained for precision, recall, and F-measure, and includes the Friedman statistic and p-value for each test (all values are less than 0.05). Classification of the algorithms according to the Friedman test on the values of recall and F-measure shows that *ICAClusterBP* achieved the best grouping. Second place was held by *GroupBPFuzzy*, while *CAClusterBP* ranks third. This classification supports the results of the external evaluation that showed the same classification order. Regarding precision, *ICAClusterBP* achieved the best results, followed by *GroupBPFuzzy* and *k-meansBP* in third place.

**Table 5:** Quality of the algorithms classification according to Friedman test (best results in bold).

Algorithm	Precision		Recall		F-Measure	
	Rank	Order	Rank	Order	Rank	Order
*ICAClusterBP* (A)	**1.9167**	**1**	**1.5**	**1**	**1.5**	**1**
*GroupBPFuzzy* (B)	2	2	2.3333	2	2.1667	2
*CAClusterBP* (C)	3.4167	4	2.5	3	2.8333	3
*k-meansBP* (D)	3.1767	3	3.8333	4	3.6667	4
*HC* (E)	4.5	5	4.8333	5	4.8333	5
p-value test	0.02546		0.00211		0.002681	
Friedman statistical test	4.30232		11.66667		10.51724	

The Wilcoxon test was used to evaluate the dominance of the results in precision, recall, and F-measure of the evaluated algorithms. Table 6 summarizes the application of the Wilcoxon test on the results of Fig 13. Black dots (●) in the rows represent dominance of the algorithm in the row over the algorithm in the column, and white dots (○) represent dominance of the algorithm in the column over the algorithm in the row. The dots (black or white) above the main diagonal have a significance level of 90%, and those below the diagonal have a significance level of 95%. Each diagonal is indicated by hyphens (-).

**Table 6:** Results of Wilcoxon test.

	Precision					Recall					F-measure				
	(1)	(2)	(3)	(4)	(5)	(1)	(2)	(3)	(4)	(5)	(1)	(2)	(3)	(4)	(5)
*ICAClusterBP* (1)	-		●		●	-		●	●	●	-		●	●	●
*GroupBPFuzzy* (2)		-		●	●		-		●	●		-		●	●
*CAClusterBP* (3)			-					-	●	●			-		●
K-*MeansBP* (4)		○		-	●	○			-		○			-	●
*HC* (5)		○		○	-	○	○	○		-	○	○	○		-

Regarding precision, it can be stated that *ICAClusterBP* has a domain with a significance level of 90% over the *CAClusterBP* and *HC* algorithms. The results for recall are better: *ICAClusterBP* outperforms *CAClusterBP*, *k-meansBP*, and *HC* with 90% significance and outperforms the *k-meansBP* and *HC* with 95% significance.

Regarding F-measure, the test shows that *ICAClusterBP* is dominant with a significance level of 90% over the *CAClusterBP*, *k-meansBP*, and *HC* algorithms. Equally, with 95% of significance level, *ICAClusterBP* outperforms the *k-meansBP* and *HC* algorithms. In second place, *GroupBPFuzzy* has a dominance of 90% of significance level over the *k-meansBP* and *HC* algorithms. The results of the Wilcoxon test, as well as the results of the Friedman test, show that *ICAClusterBP*, *GroupBPFuzzy*, and *CAClusterBP* obtain the best results in F-measure in relation to the manual evaluation performed by experts.

Grouping of BP models on large repositories can have very high computational cost and several approaches, mainly focusing on the types of information in the BPs, have therefore been proposed to address this issue. In this work, grouping of BPs is done using an iterative clustering algorithm that uses ICA lists created offline, multimodal information (textual and structural) to represent each BP, and the BBIC index to define the best solution. The experimental evaluation reveals that the groups obtained using *ICAClusterBP* are most like the ideal groups created by human experts, in comparison with groups obtained by *k-meansBP*, *HC*, *CAClusterBP*, and *GroupBPFuzzy*. All values obtained by *ICAClusterBP* in precision, recall, and F-measure are better than those obtained by the other state-of-the-art algorithms. It is notable that the value of F-measure (85.7%) of *ICAClusterBP* exceeds by 21% its best competitor (*GroupBPFuzzy*). The proposed algorithm eliminates the option of including a business process in different groups (overlapping), a key feature in increasing accuracy of the grouping and facilitating revision, by the user, of the groups formed.

## Conclusions and future work

In this paper, a new clustering algorithm is presented. The algorithm includes three phases: i) identification of the measure of similarity between BPs. This stage establishes that cosine similarity reduces intra-cluster SSE by 29% when compared with Euclidean distance used in previous work [16]. The reduction in SSE generated groups with greater cohesion (the BPs in each group have greater proximity or similarity); ii) definition of the level of strength for an ICA to evaluate a sample of groupings. The results of the evaluation of strength showed that when strength increases from 2 to 4, SSE decreases on average from 1.312 to 0.507; and iii) determination of the ideal number of groups to form. In this phase, BBIC was used to identify an ICA with alphabet v = 3 (i.e. 3 is the maximum number of clusters present in a solution) as the best value to find the grouping with the greatest proximity between the elements of each group, but this value is only related with BPs returned by each query and authors recommend using high values of alphabet (v = 6).

The algorithm has many potential applications in the grouping of text and web documents and business process models. However, the number of elements must be limited (e.g. 100) as the number of columns and rows of the covering arrays significantly increases processing time.

In the clustering process, *ICAClusterBP* showed high similarity (89.7% precision) with the grouping created by experts, exceeding *CAClusterBP* by 26%, *k-meansBP* by 21%, *HC* by 52% and *GroupBPFuzzy* by 12%. This result may result from the high refinement of the groupings since all possibilities of distribution of BPs in each group are analyzed. This process allows the identification of the grouping with lowest intra-cluster SSE. Friedman and Wilcoxon tests show that *ICAClusterBP* obtains the best results and is dominant statistically in F-measure to *CAClusterBP*, *k-meansBP*, and *HC* with a level of significance of 90%.

The results achieved in each phase of the algorithm demonstrated a substantial improvement. The algorithm makes it possible to decrease the value of the intra-cluster SSE compared with the previous version of the algorithm (*CAClusterBP*). The variation of strength t from 2 to 4, enables that at least quartets of BPs are analyzed (which can exist in a given group), thus increasing the precision of the clustering and, moreover, the groups automatically formed by the algorithm present the greatest similarity to the groups formed manually by experts.

During the evaluation, several disadvantages were found. First, it was identified that as the number of results to be grouped increases, the execution time of the grouping process also increases. Increasing the strength level in the ICAs, e.g., 5, 6 or 7 increases precision, but the execution time of the grouping process also increases. This increase is mainly due to the fact that the number of times that SSE and BBIC are calculated is higher.

The proposed BP clustering process is based on three main components: i) multimodal representation, ii) ICAs and iii) BBIC. The ICAs with strength 4 allow orienting the search of solutions and obtaining better results in comparison with other state of the art algorithms. This improvement is due to the fact that the algorithm evaluates the possible combinations of 4 in 4 BPs that should be in each group. As in other fields, a solution to a problem does not involve the exhaustive combination of all objects, but these combinations can instead be limited to smaller interactions, e.g. to 6 in software tests [55]. This fact comprises one advantage of the proposed algorithm, since a wide coverage of the possible solutions is performed in a short execution time. On the other hand, when sampling the search space only with ICAs, it is possible to get close to optimal local solutions but without reaching them. In this sense, ICAs can be seen as an exploration scheme that requires an exploitation scheme, such as the *k-means* algorithm. The latter is not included in the present work. This will be addressed in future work of the research group.

Future work will also include the development of an indexing model with additional information, such as data flow, control flow, execution flow, user information, and dates. In this model, similarity is defined by a function that weighs each type of information according to the interest of the user. This model would allow creating a semantic, automatic or semi-automatic categorization of BPs in functional categories: structural, behavioral, metadata or BP documentation [56][57]. Equally, it is planned to develop a labeling method based on the textual descriptions of all BP elements in each group. Since these descriptions define the purpose or functionality of BPs, so the returned labels would allow the user to more easily identify the functionality of each BP group. Finally, the use of mixed covering arrays to evaluate the relevance of each BP within a group will be addressed.
